# mHealth Apps for Low Back Pain Self-management: Scoping Review

**DOI:** 10.2196/39682

**Published:** 2022-08-26

**Authors:** Aki Rintala, Roy Rantalainen, Anu Kaksonen, Hannu Luomajoki, Kari Kauranen

**Affiliations:** 1 Faculty of Social Services and Health Care LAB University of Applied Sciences Lahti Finland; 2 Department of Health Zürich University of Applied Sciences ZHAW Institute for Physiotherapy Winterthur Switzerland; 3 Faculty of Social Services and Health Care LAB University of Applied Sciences Lappeenranta Finland

**Keywords:** low back pain, mobile health, mHealth, app, disability, self-management, mobile phone

## Abstract

**Background:**

The role of self-management in health promotion, as well as prevention and rehabilitation, is increasing through the use of mobile health (mHealth) apps. Such mHealth apps are also increasingly being used for self-management of low back pain (LBP), but their effectiveness has not been sufficiently explored.

**Objective:**

The aim of this scoping review was to provide an overview of the literature on self-management mHealth apps and their effects on the levels of pain and disability in people with LBP.

**Methods:**

We applied the PRISMA-ScR (Preferred Reporting Items for Systematic Reviews and Meta-Analyses extension for Scoping Reviews) methodology, including a priori research questions. A literature search was conducted in 2 databases (PubMed and PEDro) for studies published between January 1, 2015, and June 17, 2021. Interventional, cohort, or case series studies with an interventional period were included if the mHealth app included built-in self-management content, the app was used for self-management for people with LBP, and the study reported outcomes regarding pain and disability in people with LBP.

**Results:**

In total, 7 studies were selected for the review with overall 2307 persons with LBP, of whom 1328 (57.56%) were women. Among the studies (5/7, 71%) that reported the type of pain, 85% (390/459) of the participants were experiencing chronic LBP. A total of 5 different mHealth apps were identified, of which 4 contributed to a statistically significant reduction in LBP and clinically meaningful changes. Of the 7 studies, 4 (57%) used 4 different assessments for disability, of which 3 (75%) showed statistically significant improvements in the level of functional ability of participants in the experimental groups using an mHealth app with built-in self-management content for LBP.

**Conclusions:**

This scoping review supports the conclusion that people with LBP may benefit from mHealth apps that provide self-management content. However, the generalizability of the findings is limited because of heterogeneity in the pain characterization of the included participants and the intervention durations. More high-quality studies with longer follow-up periods to investigate personalized mHealth approaches are recommended for LBP self-management.

## Introduction

### Background

Low back pain (LBP) is one of the greatest concerns in health care worldwide, and it is one of the major factors in a decline in overall function [1,2]. Almost 80% of the world’s population will encounter LBP at some period in their lives, and approximately 50% will experience multiple pain periods during their lifetime [1]. In 2019, the number of prevalent LBP cases was shown to increase with age, with LBP peaking at age 45 to 54 years for both genders [3]. The origin of LBP is still unclear in the literature, but many factors contribute to its existence, such as an individual’s genome, obesity, smoking, sedentary behavior, physical labor, work posture, and excessive sitting, as well as psychological factors such as stress [3,4].

Self-management is an important treatment strategy for health promotion. Self-management is defined as any treatment method that improves or maintains health, prevents disease, and supports management of disease or disability [5,6]. The active participation of patients in their care of the symptoms or illness aiming to prevent the progression of medical conditions plays a key role in self-management [5]. Self-management content is usually focused on ergonomics, weight management, behavioral changes, and physical activity [6]. Self-management also plays a role in LBP rehabilitation, and it has been found to have a small-to-moderate effect on decreasing the levels of pain and disability in people with LBP [7].

The rapid advances in new technology have also led to a merger of new therapy approaches in rehabilitation and self-management. Mobile health (mHealth) is defined as a health and well-being mobile service that enables 2-way health-related information delivery and communication [8]. A search for mobile apps in the Apple App Store and Google Play Store at the end of 2021 revealed that >5 million different mobile apps were available, of which >100,000 apps were related to mHealth content. Furthermore, there were >500 million users worldwide using services related to mHealth apps already in 2011 [9]. These statistics show that mHealth apps are already an important part of people’s everyday life and will play an increasing role in the management of their medical care because the number of mHealth apps and their use is expected to increase in the near future.

Self-management interventions have been studied with supporting evidence as part of LBP treatment [7,10]. However, studies investigating the effects of mHealth apps with built-in self-management content are still lacking. A previous review focusing on eHealth (web-based and mobile-based) self-management programs for LBP found preliminary evidence from the subgroup meta-analysis consisting of 3 studies that supported the claim that mHealth programs may have a role in decreasing the levels of pain and disability [10]. However, the meta-analysis included only 3 randomized controlled trials (RCTs), which included mHealth apps without built-in self-management content. Only 1 systematic review has identified the commercial use of mHealth apps related to self-management for people with LBP, and it provided an overview of existing mHealth apps and their content [1]. Machado et al [1] found 61 commercially available mHealth apps targeting LBP in the Australian iTunes Store and Google Play Store. The content of the mHealth apps for LBP management was designed to provide a wide variety of exercises related to strengthening, stretching, range of motion, motor control, Pilates or the McKenzie method, yoga, tai chi, and mindfulness, or a combination of these [1]. However, it is still unclear whether such apps can be used in clinical settings.

### Objectives

Health care professionals have called for efficient tools that can be used to motivate and engage their patients in managing their LBP [1,11]. An updated overview is lacking for studies on mHealth apps with built-in self-management content and their effects on the levels of LBP and disability. New technology approaches such as mHealth apps may support traditional pain management and care and, in turn, improve patients’ abilities to self-manage their LBP in the home environment setting. The objective of this scoping review was to map the number of current mHealth apps used in research settings and to identify their effects on the levels of pain and disability.

## Methods

### Search Strategy

We conducted a literature search for studies published between January 1, 2015, and June 17, 2021, in the PubMed and PEDro databases. The literature search was also expanded to manual search, using the same search terms, in Google Scholar and reference lists of the retrieved articles. The search strategy focused on health care interventions in the form of mHealth apps and, therefore, did not include the development, construction, or evaluation of the technology itself. The search strategy contained general terms related to mHealth technology (ie, “smartphone app,” “technology,” “health app,” and “mobile health”), LBP (ie, “low back pain,” “low back ache,” “back pain,” “lumbago,” “acute lower back pain,” and “chronic lower back pain”), and self-management (ie, “self-management” and “self-care”). As the databases differed in terms of technical search options, in the PEDro database, we used the following simple search terms: “back pain mobile self,” “back pain mhealth,” and “back pain smartphone.”

The literature search was conducted by 2 reviewers (RR and AR) who screened and assessed the published articles independently. The screening was conducted with a predefined strategy, which involved first screening the titles and abstracts, followed by an assessment of the included studies based on a full-text reading by the 2 reviewers of the research team (RR and AR). A list of the studies to be included was agreed upon after resolving disagreements on eligibility through discussions. In case of disagreement, a third reviewer (AK) evaluated the studies. After including relevant studies, both reviewers also reviewed the references of the included studies for additional relevant publications.

### Inclusion and Exclusion Criteria

The literature search was limited to peer-reviewed articles published between January 1, 2015, and June 17, 2021. Studies were included in this review if they aimed to explore the use of mHealth apps for self-management in people with LBP. The definition of self-management and self-care was derived from the World Health Organization guideline on self-care interventions for health and well-being [12]: “the ability of individuals, families and communities to promote health, prevent disease, maintain health and to cope with illness and disability with or without the support of a health worker.” The scope was limited to health promotion; disease prevention and control relates to providing care to dependent persons in a rehabilitation setting.

We included any type of interventional study, cohort study, or possible case series, including an interventional period for >1 participant. Studies also needed to be published in English. Studies were excluded if the mHealth apps did not include self-management content (as described previously), did not include an interventional study period, or if participants had been diagnosed with pain other than LBP. In addition, we excluded study protocols, opinion articles, and studies other than interventional, cohort, or case series (ie, reviews, case studies, and qualitative studies).

### Data Extraction

We extracted the predefined data from the included studies after the full-text screening. Data charting was conducted first by 1 member of the research team and then reviewed by another member of the research team. Predefined data included study details (authors, year, study method, objectives, country, measurement of pain, and number of participants), mHealth details (name and content of the mHealth app), and personal (gender and age) and clinical (type of pain and duration of pain) characteristics. Type of pain was defined as acute (<6 weeks), subacute (6-12 weeks), or chronic (>12 weeks). If needed, research team members contacted the corresponding authors of the included studies for additional inquiry if the aforementioned data were not reported adequately in the original article.

### Quality Assessment

Two reviewers assessed the methodological quality of the selected studies using JBI’s critical appraisal tool [13,14]. In case of disagreement, a third reviewer evaluated the methodological quality of the selected study. Depending on the type of study methods, the number of methodological questions ranged from 11 to 13 items; for example, the maximum score for RCTs is 13 points, for case-cohort studies 11 points, and for non-RCTs 9 points. Each item was rated *yes*, *no*, *unclear*, or *not applicable*. Total and mean scores of items rated *yes* (1 point) were computed for each included study.

### Data Synthesis

Extraction data were analyzed descriptively, and if applicable, a frequency analysis was conducted for retrieved data related to study characteristics (number of participants [n]), personal characteristics (age [mean, range] and gender [n, %]), and clinical characteristics (type of pain [n, %] and pain duration [mean, range]). The effects of mHealth apps on the levels of pain and disability were calculated with a vote-counting analysis.

### Rationale

We conducted a scoping review following the JBI’s Manual for Evidence Synthesis and the PRISMA-ScR (Preferred Reporting Items for Systematic Reviews and Meta-Analyses extension for Scoping Reviews) [15,16]. For developing the research questions, we used JBI’s population, concept, and context framework to formulate the primary review questions. The primary research questions in this review are as follows:

1. How many self-management mHealth apps have been used in research settings for people with LBP?
2. What type of self-management content do mHealth apps have for people with LBP?
3. What are the effects of using self-management mHealth apps on the levels of pain and disability in people with LBP?

## Results

### Overview

**Figure 1:**
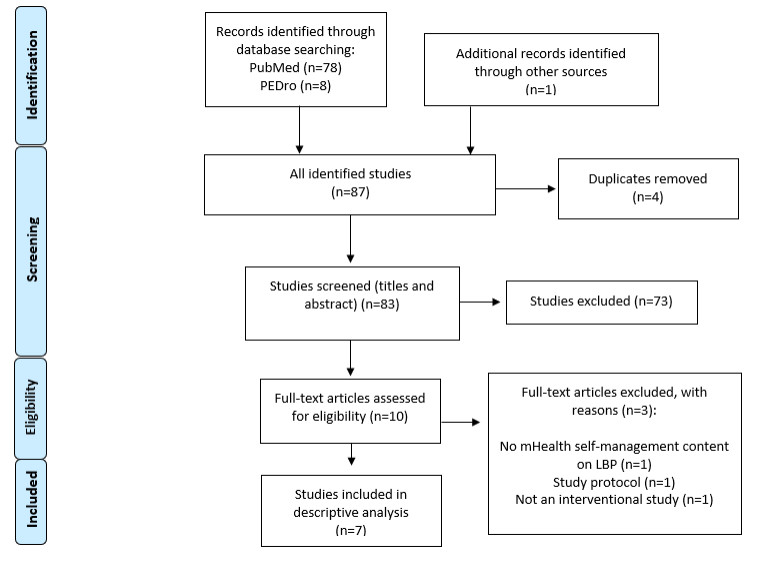
Flow chart of study selection. LBP: low back pain; mHealth: mobile health.

Of the initial 87 studies identified in the literature search, 4 (5%) duplicates were removed. Of the 83 remaining studies, 73 (88%) that did not meet the inclusion criteria after title and abstract screening were excluded. The screening of the included 10 full-text studies revealed 7 (70%) that met the inclusion criteria [17-23]. Of these 7 studies, 4 (57%) were RCTs, 2 (29%) were cohort studies, and 1 (14%) was a non-RCT (Table 1). A flowchart of the study selection is presented in Figure 1. Of the 7 studies, 4 (57%) were conducted in Europe [19,20,22,23], and 1 (14%) each in the United States, Jordan, and India [17,18,21].

**Table 1:** Study details and results of studies using a mobile health (mHealth) app for low back pain (LBP).

Study, country				Content of the study		Participants; woman, n (%), age		Type of pain: n (%); duration; medication		Name of the mHealth app		Content of mHealth app		Outcome type of the levels of pain and disability		Effects of the mHealth app on the levels of pain and disability		Vote-counting, pain;disability
**RCTs^a^**																		
		Irvine et al [21], 2015, United States		Investigating the efficacy of the mHealth app to guide user implementation of personalized strategies for LBP management and prevention (16 weeks); group 1: mHealth app; group 2: web-based email support for the LBP program; group 3: no LBP program (control)		Workers; group 1: 199 (58), age not provided; group 2: 199 (59), age not provided; group 3: 199 (63), age not provided		Not reported		FitBack		Personalized content depending on whether the person on a daily average basis is sitting, standing, driving, or lifting; general well-being; mindfulness exercises; strength exercises; stretching exercises; diary		How bad is your LBP? (6-point Likert scale); how often have you experienced LBP? (6-point Likert scale); when you experienced LBP, on average how intense was the pain? (7-point Likert scale); when you experienced LBP, on average how long did it usually last? (5-point Likert scale)		Back pain measures: group 1 level of pain decreased by 0.4 points at 16 weeks, in group 2 by 0.3 points, and in group 3 by 0.1 points; group 1 vs group 2=not statistically significant difference after 16 weeks (*P*=.17), at 8 weeks not tested; group 1 vs group 3=statistically significant differences after 16 weeks (*P*=.002), at 8 weeks not tested		+;—^b^
		Chhabra et al [18], 2018, India		Investigating the effect of using an mHealth app on pain and function in patients with chronic LBP (12 weeks); group 1: mHealth app; group 2: conventional group receiving a written prescription from the physician and a list of prescribed medicines and dosages		People with chronic LBP; group 1: 45 (not reported), 41; group 2: 48 (not reported), 41		Type of pain: chronic (>12 weeks): 93 (100); duration: group 1: 23 months; group 2: 28 months; medication: not reported		Snapcare		Personalized set of exercises based on the health status of the user using gamification: physical activity goals (eg, daily steps); home therapeutic exercises; possibility of progression based on the use of the app; focus on increasing daily life activities and increasing basic routines as independently as possible		NRS^c^ (0-10); MODI^d^ (0-50); CSS^e^ (0-25)		NRS: pain decreased by 4.0 points in group 1 and by 3.4 points in group 2 at 12 weeks; no statistically significant group differences (*P*=.23); MODI: functional ability improved by 31.9 points in group 1 and by 11.5 points in group 2 at 12 weeks; difference was statistically significant (*P*<.001); CSS improved by –9.0 points in group 1 at 12 weeks compared with baseline (*P*<.001); group differences were not tested		0;+
		Toelle et al [23], 2019, Germany		Investigating the clinical effects of a multidisciplinary mHealth app for LBP (12 weeks); group 1: mHealth app; group 2: 6 physiotherapy sessions and web-based education		Adults with LBP; group 1: 48 (73), 41; group 2: 46 (67), 43		Type of pain: chronic (>12 weeks): 94 (100); duration: group 1: 7.2 months; group 2: 6.7 months; medication: MQS^f^, group 1: 2.4; group 2: 2.8		Kaia app		Therapeutic exercises; mindfulness exercises; education regarding LBP; possibility of progression		NRS (0-11); HFAQ^g^		NRS: pain decreased by 2.4 points in group 1 and by 2.0 points in group 2 at 12 weeks; group difference was statistically significant in favor of the mHealth app group (*P*=.02); group differences were not statistically significant at baseline nor after 6 weeks (*P*>.05); within groups, both groups showed a significant decrease in pain symptoms over time (baseline vs 6 weeks and 6 weeks vs 12 weeks, all *P*<.01); HFAQ: no statistically significant differences between the groups (*P*>.05)		+;0
		Almhdawi et al [17], 2020, Jordan		Investigating the efficacy of a newly developed evidence-based mHealth app for LBP management (6 weeks); group 1: mHealth app; group 2: placebo app containing nutritional facts with no LBP management		Workers; group 1: 21 (34), 41; group 2: 20 (20), 42		Type of pain: chronic (>12 weeks): 41 (100); VAS^h^>3.0; duration: pain chronicity>3 months; medication: not reported		Relieve My Back		Education regarding LBP (general advice and instruction); home therapeutic exercises for lower back and abdominal muscles; stretching exercises for lower back and abdominal muscles		VAS (0-10); ODI^i^ (0-100)		VAS: pain decreased by –3.5 in group 1 and by –0.1 in group 2 at 6 weeks; group difference was statistically significant in favor of the mHealth app group (*P*<.001); ODI: functional ability improved by 11.5 points in group 1 and by 0.6 points in group 2 at 6 weeks; the difference was statistically significant in favor of the mHealth app group (*P*=.002)		+;+
**Cohort studies**																		
	Huber et al [20], 2017, Germany		Investigating short-term changes effected by an mHealth app for the treatment of LBP (12 weeks)		Users of the Kaia app with a history of medical treatment of back pain and no history of specific back pain; 180 (58), 34		Type of pain: acute (<6 weeks): 25 (14); subacute (6-12 weeks): 23 (13); chronic (>12 weeks): 132 (73); duration: not reported; medication: not reported		Kaia app		Therapeutic exercises; mindfulness exercises; education regarding LBP; possibility of progression		NRS (0-10)		NRS score decreased at 4 weeks by 1.3 points (*P*<.001), at 8 weeks by 1.5 points (*P*<.001), and at 12 weeks by 2.0 points (*P*=.21), with no difference between pain types (*P*>.30)		—	
	Clement et al [19], 2018, Germany		Investigating the effect on user retention and clinical outcomes of the Kaia app during development between 2 groups (24 weeks); users were grouped depending on the available version at the time of the sign-up; group 1: older version (0.x) of Kaia app; group 2: new version (1.x)		Users of the Kaia app with a history of medical treatment of back pain and no history of specific back pain; group 1: 196 (58), age not provided; group 2: 1055 (49), age not reported		Not reported		Kaia app		Pain self-management app containing several domains with the possibility of personalization; therapeutic exercises; mindfulness exercises; education regarding LBP; pain diary and self-test; chat; feedback system available for training and pain levels; possibility of progression		NRS (0-10)		Levels of pain decreased in both groups after 24 weeks: group 1 by 0.9 points and group 2 by 1.2 points with no difference between the groups (*P*=.29); within the group, the decrease in pain was statistically significant in group 1 (*P*=.008)		—	
**Non-RCT**																		
		Sandal et al [22], 2020, Denmark and Norway		Investigating the basis for recruitment and screening procedures to explore the associations between the inclusion process and questionnaires and app installation and to examine the changes in clinical outcomes (6 weeks)		People with LBP within the past 8 weeks; 51 (58), 46		Type of pain: acute (<6 weeks): 11 (22); subacute (6-12 weeks): 10 (20); chronic (>12 weeks): 30 (58); duration: not reported; medication: infrequent use: 51 (58)		selfBACK		Weekly personalized self-management plans: physical activity (number of steps per day); strength and mobility exercises; mindfulness exercises; education regarding LBP; goal setting		NRS (0-10) average past week; NRS (0-10) worst past week; pain-related disability (RMDQ^j^)		NRS average past week: pain decreased by 1.0 point (95% CI –1.6 to –0.4); NRS worst past week: pain decreased by 1.0 point (95% CI –1.6 to –0.4); RMDQ: functional ability improved by 1.8 points (95% CI –2.9 to –0.7)		—

^a^RCT: randomized controlled trial.

^b^Not available.

^c^NRS: numeric rating scale (0-10 with higher scores indicating worse pain).

^d^MODI: Modified Oswestry Disability Index.

^e^CSS: current symptom score.

^f^MQS: Medication Quantification Scale.

^g^HFAQ: Hannover Functional Ability Questionnaire.

^h^VAS: visual analog scale.

^i^ODI: Oswestry Disability Index.

^j^RMDQ: Roland-Morris Disability Questionnaire.

### Description of the Participants

The selected studies included 2307 people with LBP, of whom 825 (35.76%) were included in RCTs, 1431 (62.03%) in cohort studies, and 51 (2.21%) in a non-RCT. Of the 825 participants with LBP in RCTs, 313 (37.9%) were included in the experimental group and 512 (62.1%) in the control group. In the total sample, the mean age of the participants was 40.7 (SD 4.2; range 40-46) years, and 57.56% (1328/2307) were women. Of the 7 studies, 2 (29%) did not report age [19,21], and 1 (14%) did not report gender distribution [18].

Of the 7 studies, 5 (71%) included 390 participants with chronic LBP [17,18,20,22,23]; of these 5 studies, 2 (40%) also included people with acute (n=36) and subacute (n=33) LBP [20,22]. In total, 14% (2/7) of the studies did not report the type of pain; both these studies together accounted for the highest number of participants (n=1848) [19,21]. The exact duration of pain at baseline was only reported in 14% (2/7) of the studies (Table 1).

The main inclusion criteria for eligibility to participate in the included studies varied. The inclusion criteria were as follows: age (>18 years), ability to use a smartphone, and experiencing any level of LBP in the past days or months [17-23]. Other criteria included a minimum pain score of 4 on the visual analog scale (VAS) or ≥4 to ≥5 points on the numeric rating scale (NRS) scale [17,18,23], sufficient level of self-reported physical fitness [18-20,22], a declaration of medical treatment for back pain [18-20], or a declaration of no medical treatment for back pain [21]. In total, 57% (4/7) of the studies included participants with nonspecific LBP as a definition of perceived pain in the lower back region that was not attributable to a recognizable specific pathology (eg, infection, tumor, osteoporosis, lumbar spine fracture, structural deformity, inflammatory disorder, cauda equina syndrome, ankylosing spondylitis, spondylitis, spondylolisthesis, spondyloarthritis, spinal stenosis, or spinal disk herniation) [19,20,22,23].

### Methodological Quality

The overall methodological quality of the studies is described in Multimedia Appendix 1 [17-23]. The methodological quality of the RCTs (n=4) varied from fair to good. A general drawback was the blinding of outcome assessors and reporting of the outcome assessment reliability procedures. This was expected because the blinding procedure is difficult to achieve (outcome assessors as well as participants) in these types of interventional studies. There were similar issues with the cohort studies (n=2) because they did not clearly report confounding factors or strategies thereof; furthermore, strategies for completion were not always reported.

### Self-management Content of mHealth Apps

This review identified 5 different mHealth apps containing self-management content for people with LBP (Table 1). Content varied based on the app and the level of reporting in each study, but commonalities were personalization, increasing as well as monitoring daily life activities and physical activity, targeted home exercises (strengthening and stretching), mindfulness training, and education regarding LBP. Of the 7 studies, 4 (57%) reported the possibility of personalizing the mHealth app content in the apps (Kaia app, Snapcare, FitBack, and SelfBACK) [18,19,21,22], and 3 (43%) studies reported a possibility for participants to build a training progression in 2 apps (Kaia app and Snapcare) [18,20,23]. More specified description of the app versions, use, training progression, and personalization of the content was not reported. We have listed the main content for each app in the following paragraphs.

Kaia app was used in 43% (3/7) of the studies [19,20,23]. The app was designed to include three domains: (1) back pain–specific education, (2) physiotherapy (a pool of 145 exercises adapted to the user’s fitness level), and (3) mindfulness exercises. The app also contained units dedicated to breathing techniques, body scan, and progressive muscle relaxation. Users had the possibility to optimize the content on a daily basis depending on their status with regard to knowledge, practice, and progress. Educational content was focused on general pain–related and back pain–specific education (overall, there were 30 different educational units).

The remaining (4/5, 80%) apps—SelfBACK [22], FitBack [21], Relieve My Back [17], and Snapcare [18]—were used in individual studies.

SelfBACK contained weekly user-tailored general physical activity, strengthening and flexibility exercises, and patient education [22]. Other minor units in the app were a goal-setting tool, audio mindfulness exercises, pain-relieving exercises, and general information about LBP.

FitBack included a self-tailored cognitive behavioral approach where the main focus was to monitor the levels of pain and activity and to provide LBP-related in-app text and video messages [21]. The content was administered based on the user’s job type (sitting most of the day, standing most of the day, driving most of the day, or lifting most of the day). Other units in the app were unlimited access to pain management education, instructional videos on strengthening and stretching exercises tailored based on the job type, and live web-based streaming instructions on ergonomics and exercises.

Relieve My Back contained general advice and instructions to conduct exercises at work and home. Office-based exercises were focused on stretching, whereas home-based exercises in the evening included strengthening exercises, both focusing on the lower back and abdominal muscles [17]. The app also provided prompts to remind the users to take a walk break, check their posture, and perform the exercises.

Snapcare included 2 main units: monitoring the levels of daily activities and monitoring the user’s symptomatic profile [18]. Daily activity goals, including back and aerobic exercises, were developed based on the user’s health status, activities of daily living, and daily activity progress.

### mHealth Interventions

#### Interventions in RCTs

The training periods ranged from 6 to 16 weeks (Table 1). Participants used solely a smartphone-based mHealth solution in their interventions. Regarding the environmental settings of the mHealth interventions, all interventions were applied in an at-home environment. mHealth interventions were compared with either web-based email support for LBP and no rehabilitation [21], conventional training [18], physiotherapy and web-based education [23], or a placebo app containing nutritional facts without LBP self-management [17].

#### Interventions in the Cohort Studies and Non-RCT

The cohort studies [19,20] and non-RCT [22] followed participants’ use of mHealth apps for 24 weeks, 12 weeks, and 6 weeks, respectively (Table 1). Of these 3 studies, 2 (67%) focused on user retention and clinical outcomes of pain [19,22], and 1 (33%) investigated short-term changes in LBP using an mHealth app [20].

### Assessments of Pain and Disability

The clinical outcomes of pain are described in Table 1. Of the 7 studies, 6 (86%) used either the VAS or the NRS to measure self-reported LBP. Of these 6 studies, 1 (17%) used 4 items, including 5- and 7-point Likert scales, to determine the current level, frequency, intensity, and duration of LBP [21], and 4 (67%) determined the level of disability related to LBP using the Modified Oswestry Disability Index, Hannover Functional Ability Questionnaire, Oswestry Disability Index, or Roland-Morris Disability Questionnaire [17,18,22,23].

### Effects of Using mHealth Apps for LBP Self-management on the Level of Pain

#### RCTs

Of the 4 RCTs, 3 (75%) reported statistically significant changes in decreases in the level of LBP in favor of the mHealth app group compared with the physiotherapy and web-based education [23], web-based email support group or no training [21], or placebo (nutritional) [17] groups (Table 1), whereas 1 (25%) did not show statistically significant changes in the level of pain between the groups when mHealth app interventions were compared with conventional training [18].

#### Cohort Studies and Non-RCT

All (3/3, 100%) of the studies reported a statistically significant decrease in LBP in participants using mHealth apps for 6 weeks [22], 4 and 8 weeks [20], and 24 weeks [19]. Of the 3 studies, 1 (33%) found a decrease in the level of pain at 12 weeks, but this was not statistically significant when observing the main effect of time for the pain ratings [20].

### Effects of Using mHealth Apps for LBP Self-management on the Level of Disability

The level of disability was assessed in 57% (4/7) of the included studies using 4 different disability assessments (Table 1). Of the 3 RCTs, 2 (67%) reported a statistically significant change in improving functional ability compared with conventional training [18] or placebo (nutritional) group [17], whereas 1 (33%) did not report statistically significant differences in functional ability between mHealth app intervention and traditional physiotherapy [23]. The non-RCT showed a statistically significant improvement in functional ability for participants in the mHealth intervention group over 6 weeks [22].

## Discussion

### Principal Findings

This scoping review found 5 mHealth apps for LBP self-management that were used in research settings (n=7 studies) to investigate their effects on the levels of LBP and disability. The majority of the studies reported promising evidence of the effects of the mHealth apps on decreasing the levels of pain (6/7, 86%) and disability (3/4, 75%) when the focus of the studies was on self-managing LBP. However, heterogeneity was observed across the studies regarding the mHealth apps, the type and duration of pain across participants, and the comparison groups, all of which diminish the possibility of a robust conclusion in this review. Despite these heterogeneity aspects, some general conclusions can be drawn.

When we view our findings regarding the content of the mHealth apps, our analyses were similar to those presented in 2 previous studies [1,10]. Most of the content included therapeutic exercises focusing on strength, mobility, and mindfulness. Our review identified only 5 mHealth apps that were used in a research setting, whereas the systematic review by Machado et al [1] provided a general overview of existing commercial apps that included 61 different apps. The review by Du et al [10] used 3 mHealth studies in the subgroup meta-analyses. The reason for narrowing our focus and including only studies involving mHealth self-management apps in our review was to ascertain the current state of these apps to provide preliminary scientific support to self-manage the levels of LBP and disability when using mHealth apps with self-management content. We also excluded studies if the mHealth apps did not include built-in, self-management content, which makes the overview of this scoping review more targeted to such mHealth apps.

Our review showed supporting evidence that mHealth apps targeting self-management may have their place as an additional tool in LBP self-management in a home environment setting. This was also supported by a previous meta-analysis of 3 studies [10]. However, Du et al [10] also included in their meta-analysis a study on an app that was targeted to only report daily data without specific built-in self-management content in the app itself [24]. For providing such services in clinical or home environment settings, it must be taken into account how clinically meaningful the results are for the level of pain. The included studies assessed the level of pain mostly using an 11-point Likert scale assessment (eg, the VAS and the NRS) that is commonly used in clinical practice because of its ease of use as well as evidence of the validity and reliability of its measures [25]. Another review also pointed out that when comparing the measurement properties of the VAS and the NRS, no evidence was provided to indicate that one was superior to the other in the measurement of LBP [26].

For the included RCTs in our review that reported statistically significant differences in favor of the mHealth group in the level of pain measured with the NRS, the changed values varied from –2.0 to –4.0 points, which can be considered a minimal clinically important change according to a previous study reporting a minimum threshold of –2.0 points or a percentage value of –33%, each of which was associated with better improvement in chronic musculoskeletal pain intensity [27]. For the VAS, an included RCT reported a decreased value of 3.5 points [17], which also can be considered within the threshold (30 mm) of a minimal clinically important difference score that was reported in a previous study investigating the levels of minimal clinically important difference scores on the VAS to measure pain [28]. Given that all included studies reported the levels of pain to be above the minimal clinical threshold, we may carefully conclude that mHealth interventions targeted at self-management may achieve a clinically meaningful change in the level of pain within intervention periods lasting from 6 to 16 weeks. Although this is a promising finding, more studies are required to investigate whether such clinically meaningful change is detectable and sustainable over a much longer period of time.

Another aspect of investigating the use of an mHealth self-management app for LBP was to identify its effects on the level of disability. In our review, this was measured in clinical trials (3 RCTs and 1 non-RCT) showing that, of the 4 studies, 3 (75%) did show a statistically significant change in improving functional ability. It seems that using mHealth self-management apps in a home setting may improve the functioning of patients with LBP. This could be a game changer, especially given the fact that the functional ability of patients with LBP is usually affected by anxiety and fear [29]. That said, mHealth self-management apps could provide help for these patients to decrease the worries related to LBP, in addition to providing clinical care. However, more research is needed to investigate the relationship between mHealth app content and the level of disability to confirm these early findings using more sophisticated analyses (eg, meta-analysis and meta-regression).

Achieving optimal management of LBP also requires the patient to play an active role and participate in the treatment. This was highlighted in another review that pointed out several aspects with regard to the patients wishing for more patient-centered care mapping the desirable characteristics of health care professionals, patients’ information needs, aspects of care, and barriers to care [30]. From these key elements, the mHealth approach could facilitate some factors related to care, where Chou et al [30] reported that participants wished for more holistic, personalized, emotionally supportive, and encouraging health care as well as the need for continuity of care. mHealth self-management apps could support this when providing an extension of care alongside clinical care. In addition, participants wished to have more information available related to their diagnosis and cause of pain [30]. This was also part of the content of the mHealth apps included in our review.

When we explore the use of mHealth apps in LBP self-management, we should also think critically about the patient for whom this may be more feasible. Although our review consisted of studies involving >2000 participants, almost half (3/7, 43%) of the studies reported very poorly the duration of pain at baseline in people with LBP. In addition, among the studies (5/7, 71%) that reported the type of pain, 85% (390/459) of the participants were experiencing chronic LBP. Therefore, the majority (5/7, 71%) of the included studies that reported the type of pain included participants with chronic pain; even so, it is still too early to conclude whether mHealth apps are beneficial for a certain type of LBP when they are targeted at self-management of the symptoms. It seems that mHealth apps may be an alternative method alongside individual treatment strategies in coping and dealing with pain. However, a question mark remains over the timing and use of mHealth apps in LBP to maximize support for patients.

The methodological quality of the included trials varied from fair to good. Overall, none of the included studies showed a poor methodological quality, which can be considered a promising finding. The included RCTs had mainly inadequate reporting related to treatment allocation, blinding of participants, and blinding of outcome assessors. Given the types of interventions, the difficulty of blinding participants or outcome assessors can be considered understandable. However, the reliability of the selected outcome assessments was only adequately reported in 50% (2/4) of the RCTs. The RCTs had sample sizes ranging from 20 to 199 participants in the experimental groups, with 75% (3/4) of the studies including relatively low sample sizes (<50 participants), which may lower statistical power and hinder the vote-counting analysis of this scoping review. With regard to the other included studies, mainly the cohort studies, the primary issues concerned insufficient reporting of possible confounding factors. Finally, the methodological quality assessment did not assess the existence of possible participation in other therapies (cointervention bias), which, if not reported, can be considered a confounding factor with regard to drawing conclusions about the effects of mHealth apps on our outcomes of interest. Furthermore, this should be reported more clearly and taken into account when assessing the effects of mHealth on the levels of pain and disability in people with LBP.

This review includes some limitations. First, a selection bias cannot be ruled out during the literature screening procedures of this review. Studies were excluded if they did not explicitly report an mHealth app–based intervention in the title or abstract. Second, we only included studies that were published after January 1, 2015. It is possible that older studies have been published that should have been included in this review. However, this decision was made based on a previous review by Du et al [10] and also based on our presearch to identify proper keywords for our search strategy. Third, the generalizability of the results is limited because a few studies that included a high sample size did not report the duration of pain or the type of pain (acute, subacute, or chronic). Another aspect that limits the generalizability was the lack of reporting to understand patients’ acceptance of using an mHealth app for LBP self-management, as well as the intensity and frequency of use.

### Future Study Recommendations

More large-scale RCTs investigating the effects of mHealth apps in LBP self-management are needed with a comparison of similar treatments. In our review, all (4/4, 100%) included RCTs were relatively heterogeneous, precluding a comparison of treatments. In addition, the duration of the included interventions in the RCTs ranged from 6 weeks to 16 weeks and in the cohort studies from 12 to 24 weeks. We cannot yet draw conclusions regarding long-term effects of using mHealth apps for LBP self-management, and the feasibility of the apps for a targeted type of pain is still not fully explored. Therefore, we require longer follow-up periods (>16 weeks) to investigate the effects as well as clinically meaningful change over time. Another important clinical aspect for future studies is to measure the role of mHealth apps in behavioral changes in LBP because mHealth apps may provide additional support to patients to overcome barriers related to LBP and provide further support in home environment settings in addition to clinical care.

### Clinical Implications

Current research supports the use of mHealth as an additional tool alongside traditional care. Such mHealth apps may provide additional support for clinical care targeted to provide support in home environment settings for LBP. However, this review was limited to the information provided in each study for the content of the apps. It is possible that the app versions and the content of each app have been developed further. In addition, the use of mHealth apps for a longer period of time may require additional costs, and the apps may not be publicly available worldwide, which may narrow the targeted need for such mHealth apps. Future studies should also report more specific details bearing in mind the clinical use of the apps.

### Conclusions

Promising results were found for mHealth self-management apps on decreasing the levels of pain and disability in people with LBP. However, more high-quality RCTs with longer study periods are needed to provide further evidence on whether mHealth apps have longer-term effects on LBP self-management in home environment settings.

## Appendix

Multimedia Appendix 1 The methodological quality of the included studies that used a mobile health app designed for low back pain.

## Abbreviations

LBP: low back pain
mHealth: mobile health
NRS: numeric rating scale
PRISMA-ScR: Preferred Reporting Items for Systematic Reviews and Meta-Analyses extension for Scoping Reviews
RCT: randomized controlled trial
VAS: visual analog scale
