# Supplementary material for: mHealth Apps for Low Back Pain Self-management: Scoping Review
Source: JMIR Mhealth Uhealth. 2022 Aug 26;10(8):e39682. doi: 10.2196/39682 (PMC9463614; doi:10.2196/39682)
Supplement: Multimedia Appendix 1 [file mhealth_v10i8e39682_app1.docx]

**Multimedia Appendix.**

This is a Multimedia Appendix to a full manuscript published in JMIR Mhealth Uhealth.

For full copyright and citation information see (JMIR Mhealth Uhealth 2022;10(8):e39682) doi: 10.2196/39682

**Table 1.** The methodological quality of the included randomized controlled trials that used a mobile health app designed for low back pain.

| Study | Scoring (yes, ?, or no) | 1: Randomization | 2: Treatment allocation | 3: Group similarity | 4: Blinding of participants | 5: Blinding of treatment delivery | 6: Blinding of outcome assessors | 7: Treatment groups treated identically | 8: Follow-up | 9: Participants analyzed in groups | 10: Outcome assessment similarity | 11: Outcome assessment reliability | 12: Statistical analysis | 13: Trial design or other deviations |
| --- | --- | --- | --- | --- | --- | --- | --- | --- | --- | --- | --- | --- | --- | --- |
| Irvine et al [21], 2015 | 7/2/4 | Y^a^ | ?^b^ | Y | N^c^ | N | ? | Y | ? | Y | Y | ? | Y | Y |
| Chhabra et al [18], 2018 | 12/1/0 | Y | Y | Y | N | Y | Y | Y | Y | Y | Y | Y | Y | Y |
| Toelle et al [23], 2019 | 8/2/3 | Y | N | Y | ? | N | ? | Y | Y | Y | Y | ? | Y | Y |
| Almhdawi et al [17], 2020 | 11/0/2 | Y | Y | Y | Y | Y | ? | Y | Y | ? | Y | Y | Y | Y |

^a^Y: yes.

^b^?: unclear.

^c^N: no.

**Table 2.** The methodological quality of the included cohort studies that used a mobile health app designed for low back pain.

| Study, year | Scoring (yes, ?, or no) | 1: Group similarity and recruitment | 2: Exposure similarity | 3: Exposure validity and reliability | 4: Confounding factors | 5: Strategies for confounding factors | 6: Baseline outcome situation | 7: Outcome assessment reliability | 8: Follow-up time | 9: Follow-up completion | 10: Strategies for completion | 11: Statistical analysis |
| --- | --- | --- | --- | --- | --- | --- | --- | --- | --- | --- | --- | --- |
| Huber et al [20], 2017 | 5/2/1 | N/A^a^ | N/A | Y^b^ | ?^c^ | N^d^ | N/A | Y | Y | N | Y | Y |
| Clement et al [19], 2018 | 7/0/3 | Y | Y | Y | ? | ? | N/A | Y | Y | Y | ? | Y |

^a^N/A: not applicable.

^b^Y: yes.

^c^?: unclear.

^d^N: no.

**Table 3.** The methodological quality of the included non-randomized controlled trial that used a mobile health app designed for low back pain.

| Study, year | Scoring (yes, ?,or no) | 1: Clear cause and effect | 2: Comparison of similarity | 3: Participants included in any comparisons receiving similar treatment | 4: Control group | 5: Pre- and postintervention assessments | 6: Follow-up | 7: Outcome assessment similarity | 8: Outcome assessment reliability | 9: Statistical analysis |
| --- | --- | --- | --- | --- | --- | --- | --- | --- | --- | --- |
| Sandal et al [22], 2020 | 7/1/1 | Y^a^ | Y | Y | N^b^ | Y | Y | Y | ?^c^ | Y |

^a^Y: yes.

^b^N: no.

^c^?: unclear.
